# Supplementary material for: In Vitro and In Vivo Therapeutic Potential of 6,6′-Dihydroxythiobinupharidine (DTBN) from Nuphar lutea on Cells and K18-hACE2 Mice Infected with SARS-CoV-2
Source: Int J Mol Sci. 2023 May 5;24(9):8327. doi: 10.3390/ijms24098327 (PMC10179516; doi:10.3390/ijms24098327)
Supplement: Supplementary file 1 [file ijms-24-08327-s001.zip › ijms-2327060-supplementary.docx]

**Supplementary Materials**

Table S1. Mean area ratio (tissue / virus) = **60.40**; Standard deviation of area ratio (tissue / virus) = **67.15.**

Experiment 14 **Control Untreated:** Area ratio = 4.17

| TISSUE | | VIRUS | |
| --- | --- | --- | --- |
| Intensity | Area (pixels) | Intensity | Area (pixels) |
| 154145816 | 819831 | 36470742 | 196751 |

Experiment 15 **Control Untreated:** Area ratio = 28.61

| TISSUE | | VIRUS | |
| --- | --- | --- | --- |
| Intensity | Area (pixels) | Intensity | Area (pixels) |
| 149144717 | 811032 | 4639155 | 28349 |

Experiment 16 **Control Untreated** Area ratio = 18.78

| TISSUE | | VIRUS | |
| --- | --- | --- | --- |
| Intensity | Area (pixels) | Intensity | Area (pixels) |
| 173645026 | 920092 | 11436765 | 48998 |

Experiment 17 **Control Untreated** Area ratio = 11.79

| TISSUE | | VIRUS | |
| --- | --- | --- | --- |
| Intensity | Area (pixels) | Intensity | Area (pixels) |
| 118967366 | 705531 | 9559044 | 59834 |

Experiment 18 **DTBN treated:**: Area ratio = 56.90

| TISSUE | | VIRUS | |
| --- | --- | --- | --- |
| Intensity | Area (pixels) | Intensity | Area (pixels) |
| 203338819 | 1242762 | 6392038 | 21842 |

Experiment 19 **DTBN treated**: Area ratio = 184.86

| TISSUE | | VIRUS | |
| --- | --- | --- | --- |
| Intensity | Area (pixels) | Intensity | Area (pixels) |
| 142775393 | 784196 | 632860 | 4242 |

Experiment 21 **DTBN treated**: Area ratio = 117.71

| TISSUE | | VIRUS | |
| --- | --- | --- | --- |
| Intensity | Area (pixels) | Intensity | Area (pixels) |
| 146269205 | 810683 | 1268899 | 6887 |

Lungs of 4 mice and 3 lungs from DTBN treated mice (see materials and methods) were scanned and analyzed independently. The unpaired t- test of the area ratios was computed, showing a significant difference between the control and treated two groups (p value = 0.0215).

Figure S1. H&E stained lung tissue of normal, untreated and uninfected mice.

**
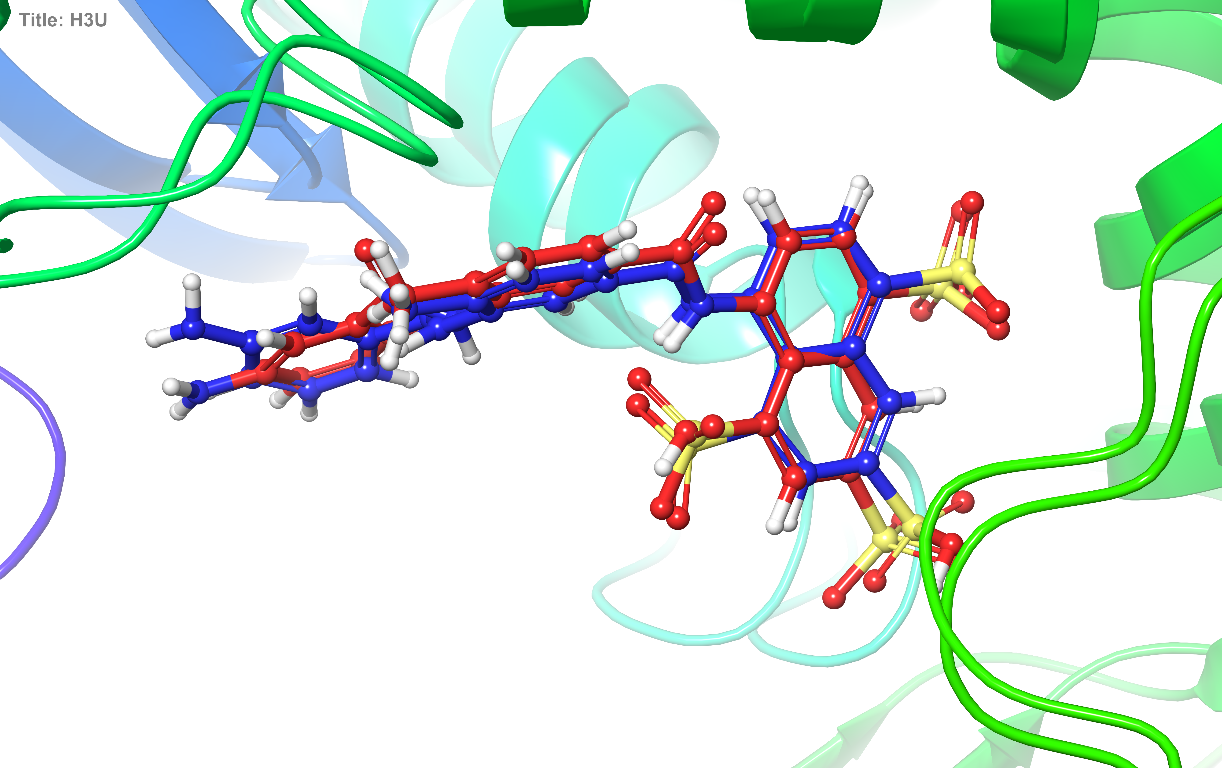
**

**Figure S2.** Superimposed image of Native Crystal ligand of Suramin bound to RdRp (Red) with Re-Docked Suramin at the active site of RdRp (Blue).

**
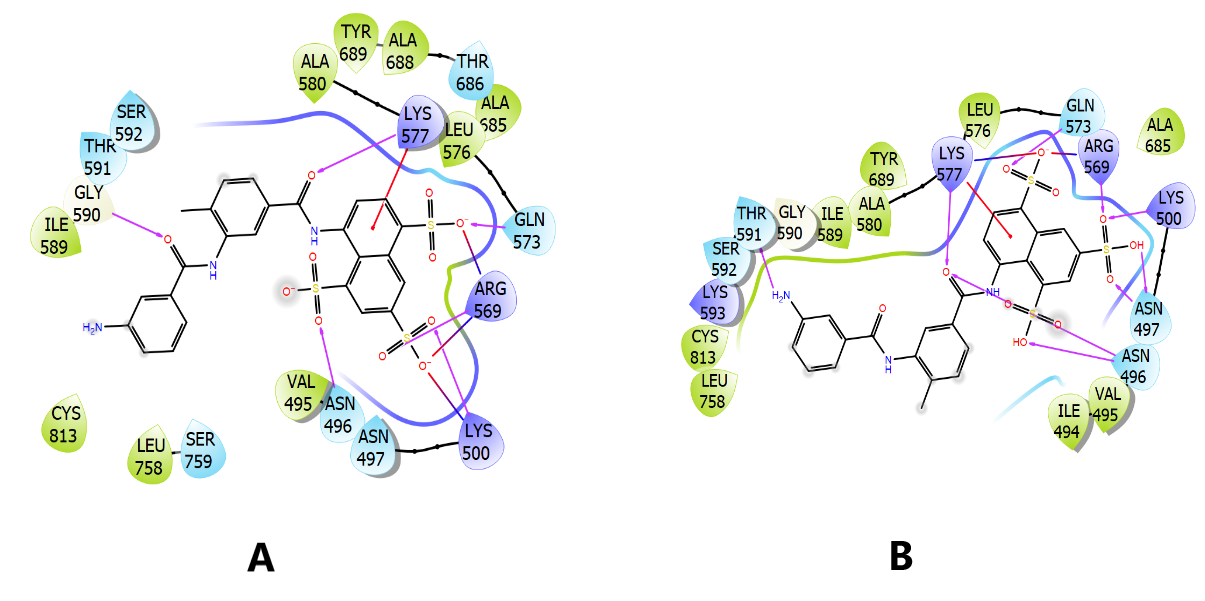
**

**Figure S3.** 2D interaction Diagram of (A) Native Crystal Structure Bound Suramin (B) Re-Docked pose of Suramin to Crystal Structure.

*
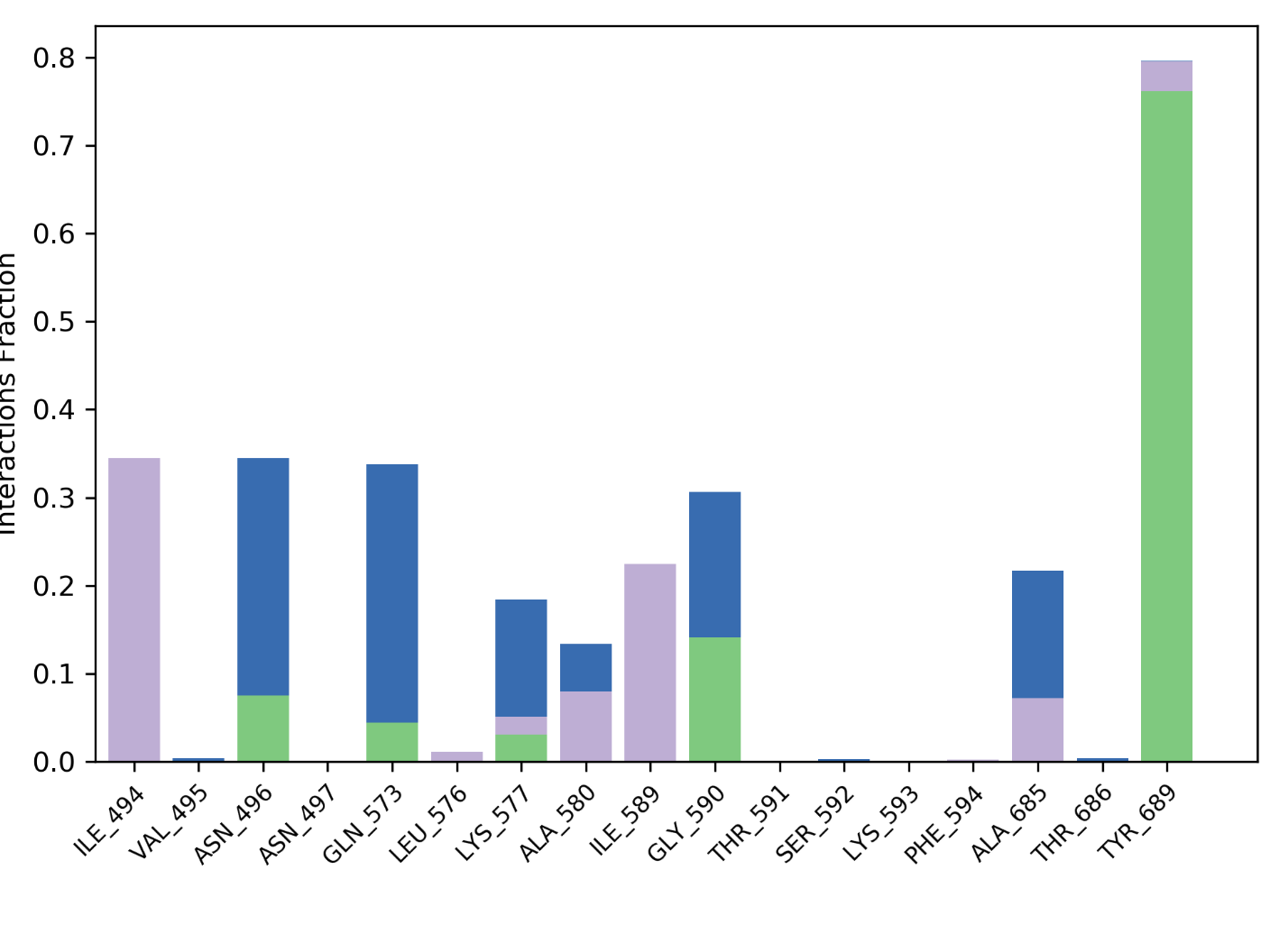
*

**Figure S4.** The interaction of DTBN with specific amino acid residues of RdRp during MD simulation.


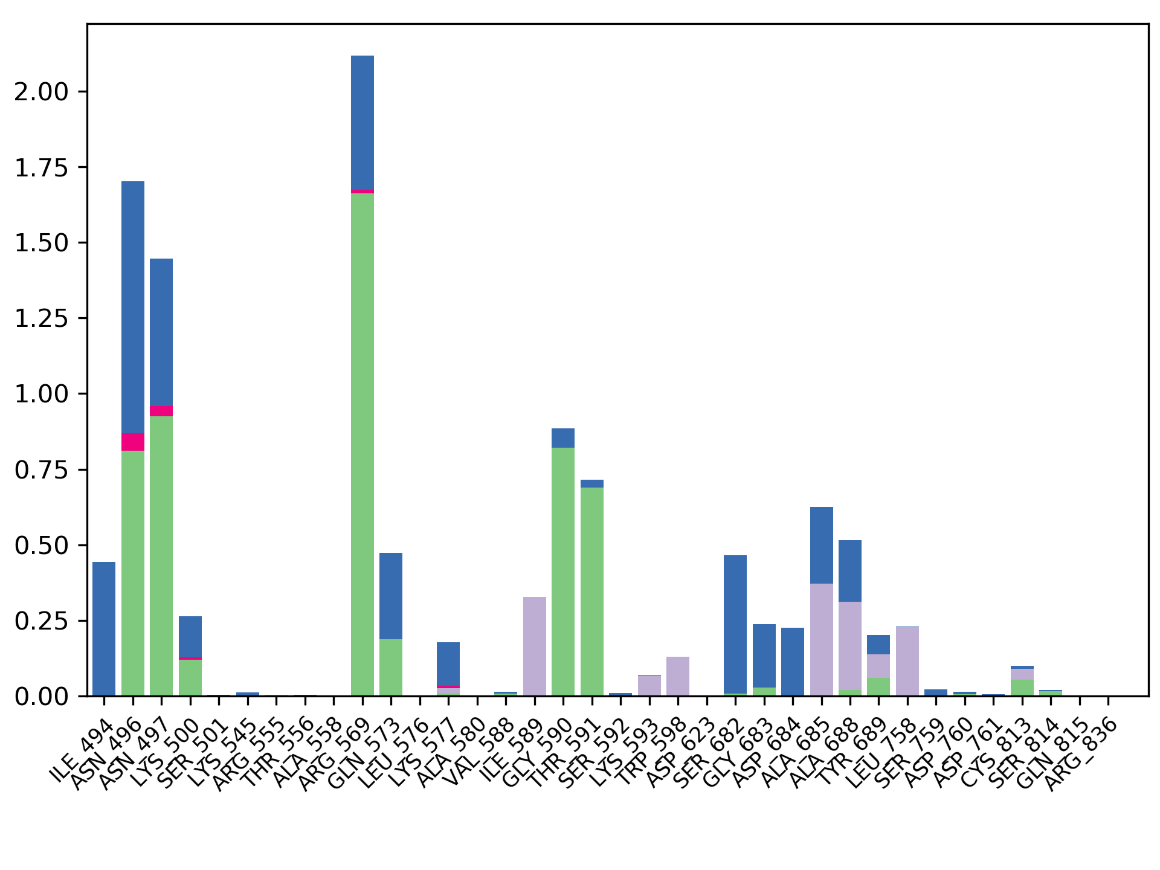


**Figure S5.** The interaction of Sumarin with specific amino acid residues of RdRp during MD simulation.
